# Targeting the undruggable: an interview with Leila Akkari on fighting cancer by rewiring cells

**DOI:** 10.1038/s42003-021-02092-3

**Published:** 2021-05-07

## Abstract

Leila Akkari began her independent career in 2017 as an Assistant Professor at the Netherlands Cancer Institute in Amsterdam after working at Memorial Sloan Kettering Cancer Center in NYC. Two years ago, she was selected as one of the junior members of the Oncode Institute, a virtual group of cancer research labs based on the Netherlands. In this short Q&A, she tells us about her research and how her diverse background has helped her as a scientist. Dr. Akkari also shares some great pointers on the biggest hurdles women in STEM face and tips to overcome them.

**Figure:**
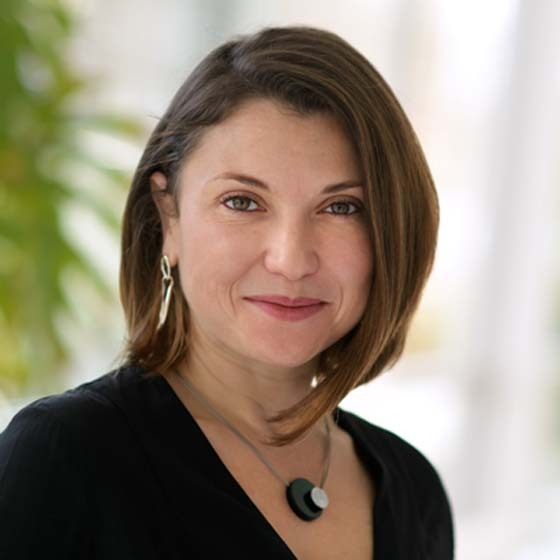
Leila Akkari

Please tell us about your research interests.

Since my early training, my scientific interests have been centered around understanding how external cues regulate cancer malignancy, with the realization that despite the groundbreaking discoveries of oncogenes and tumor suppressors, effective therapeutic options are still limited for too many cancer types. Because tumor progression relies on more than genetic/genomic alterations, and cancers evolve within a complex tissue and tumor microenvironment (TME), I have been drawn to deciphering the dynamic regulation of cells composing the TME.

To answer these questions, our lab research focuses on the role of myeloid cell populations, often the most abundant non-cancerous cell types in solid tumors, to identify vulnerabilities in the heterotypic communication they establish with cancer cells that can be targeted therapeutically. We study the microenvironment-mediated mechanisms of tumor outgrowth, therapeutic resistance and recurrence in brain and liver malignancies. In particular, we investigate the acquired resistance mechanisms resulting from dynamic alterations in the activation and recruitment of macrophages and their mediators in response to standard of care treatment. Our model systems of choice are diverse, to say the least! We utilize genetically engineered murine models of glioma and hepatocellular carcinoma combined with lineage tracing system, ex vivo cocultures and a plethora of functional assays to unravel the hijacked functions of resident and infiltrating macrophages and other myeloid cells in sheltering tumor cells. As brain and liver tumors are infamous for their lethality and limited response to immunotherapy, while being abundantly infiltrated by myeloid cells, I believe this is where we could make the most impact in the fundamental understanding of the TME regulation of primary cancers, to eventually harness these cells therapeutically.

What unknown in cancer research are you most excited about uncovering?

“Targeting the undruggable” has been an expression often used in cancer research related to hitting the most mutated oncogenes as Myc, Ras and others. Work from several brilliant labs have now tackled this challenge and are on the way to implement these therapies in the clinic.

In the TME field, these ‘undruggables’ could be seen as myeloid subsets-not because we do not know how to “drug” them, but we yet have to understand when and in which context we should.

This bottleneck lies in many aspects of the fascinating biology of these cells; their diversity, complexity and mainly, their exquisite plasticity. We need to develop ingenious systems to study these cells, particularly when isolating them from patient samples, so we can rewire their hijacked intrinsic tumor-killing phenotype into anti-cancer functions. This is why mobilizing innate leukocytes in cancer treatment, particularly in combination therapy, is my “moonshot”. Indeed, while T-cell centric immunotherapies have revolutionized modern treatments of a subset of cancers, this approach does not work in too many tumor types. The growing interest in the TME field to harness other components of the cancer immune contexture, myeloid cells included, have yet to bear fruits in the clinic despite the enormous potential of these cells. It will likely take a village, not just one lab! But thankfully, brilliant research groups across the world are tackling this question on multiple fronts, so we can altogether bring myeloid cell targeting to the front of cancer therapies in the coming decade.

What are your predictions for your field in the near future, given the huge increase in interest in immuno-oncology?

I believe we have seen only the beginning of immuno-oncology potential as an anti-cancer therapy. The next decade will be focused on identifying which cancer types and subsets will benefit from particular immune-oncology drug combinations with current standard of care treatment, and the mechanisms of resistance that may be predictable and targetable. In light of the considerable progress we are making in mapping the uncharted territories of the heterogeneous myeloid cell landscape, these cells will likely be new actors of immunotherapy. Moreover, new approaches in -omics and imaging will hold significant value to predict which cancers subtypes will respond to specific immunomodulation, so better therapies and care can be applied to subpopulation of patients. With what I anticipate will be a decade of discoveries focused on myeloid cells’ functional rewiring in tumors, I anticipate that these advances will complement the current T-cell centric therapies already in place. But of course, we still have a lot of work to do in that area! Critically, pairing up with clinicians and pharmaceutical industries will be central to bring new immune-oncology approached from bench to bedside.

With roots in Africa and the Middle East and having lived in different parts of the world, how has your diverse personal background influenced you as a scientist?

I see my personal background as one of my strongest assets, for all it has brought me as an individual and as a scientist. My parents were both immigrants when they met in France in the mid-70s, where they had to start their lives from scratch, coming from very modest families. My father was escaping the Lebanese civil war at the time and my mother had decided to leave her native Tunisia, with the hope to have better chances at a career in France. However, they never forgot their roots and raised me and my siblings as proud children of mixed backgrounds, truly connected to our origins and traditions while embracing the chance of growing up as French citizens (which they themselves were not until we were born!).

This medley of cultures, the connections to our large families abroad, our sense of belonging have all guided me in building a home anywhere I was. It drove my curiosity of the world, making me eager to discover new places, ways of life and it strengthened the friendships I made during my life abroad. As a researcher, the most valuable add-on is that I have been exposed to different scientific environments, with all kinds of interactions, distinct communities and approaches to conduct science. It has been invaluable to shape me as the scientist I have become. Most importantly, this diverse background drove my urge for inclusiveness and need for diversity in my surrounding. Without doubt, it contributed to making each workplace I had part of my second home.

I also believe it helped me build the same sharing and supportive environment in my own lab, making sure the brilliant people I have in the group feel safe, happy and connected to each other. As a scientist, this is what we should all aim for in order to excel in our research goals, but also to know we can rely on each other in joyful or more dire times.

What do you think is the biggest hurdle for women in STEM? How have you dealt with it?

Women in science are expected to do it all without much help from their surroundings, or to be honest, from society. They are supposed to always be available, to handle the organizational aspect of the job much more than what men often do, to successfully manage their careers, travel to international conferences, be dedicated teachers etc… all the while being present at home and being the committed mothers and wives.

I am not saying we cannot do it all - I have several examples around me of brilliant women in STEM who do - I mainly think that more support should be put in place to assist our professional ambitions and careers without having to sacrifice family life. Daycare is a must-have in the workplace and ideally, in conference sites as well, to not limit our international exposure. In general, it is the society mindset, the implicit bias that I believe hurts our achievement the most (both in men and women!), and that is not an easy thing to correct, but we should strive to.

I have been very lucky in that my scientific field has been pioneered by incredible female leaders, Mina Bissell, Joanne Brugge, Lisa Coussens and my own mentor Johanna Joyce. They inspired my ambitions and their examples encouraged me to pursue my scientific dreams without thinking too much about any hurdles that may arise - not many have; again, I have been lucky. The very few times I heard comments like “you will get this position/grant/opportunity because you are a woman, it is positive discrimination”, it never deterred me from doing and loving my job, it just pushed me even further to be and do my best professionally.

I believe that representation matters. We need more female role models to show the next generations of women in STEM that they can reach the same goals, we need to empower them, give them opportunities whenever possible and nurture their ambitions.

What is your favourite type of immune cell?

Without any doubts, macrophages! These cells can pretty much do anything, migrate to any tissues on demand, eat other cells, present antigens, live long in tissue, adjust their functions almost instantly to adapt to their surrounding cues…They are incredibly fascinating to study, but also challenging to rewire! Once we understand their enormous plasticity and their context-dependent specificity, I believe they will become invaluable tools to educate our immune system in anti-cancer treatment.

*This interview was conducted by Associate Editor Eve Rogers.*

